# Modified parylene-N films as chemical microenvironments for differentiation and spheroid formation of osteoblast cells

**DOI:** 10.1038/s41598-020-71322-1

**Published:** 2020-09-16

**Authors:** Tae-Hun Kim, Jong-Sook Lee, Hanhee Jo, Yusun Park, Mijin Yun, Zhiquan Song, Jae-Chul Pyun, Misu Lee

**Affiliations:** 1 Department of Materials Science and Engineering, Yonsei University, 50 Yonsei-Ro, Seodaemun-Gu, Seoul, 03722 Republic of Korea; 2 Division of Life Sciences, College of Life Science and Bioengineering, Incheon National University, 119 Academy-ro, Yeonsu-gu, Incheon, 22012 Republic of Korea; 3 Department of Nuclear Medicine, Severance Hospital, Yonsei University College of Medicine, 50 Yonsei-ro, Seodaemun-gu, Seoul, 03722 Republic of Korea

**Keywords:** Biomaterials - cells, Biomaterials

## Abstract

In this work, the influence of parylene N film on the spheroid formation of osteoblast-like cells (MG-63) was determined and compared with that of high-hydrophilicity microenvironments, such as hydrophilic culture matrix and ultraviolet-treated parylene N film. To elucidate the change in cell properties due to the microenvironment of parylene N film, global gene expression profiles of MG-63 cells on parylene N film were analyzed. We confirmed the upregulated expression of osteoblast differentiation- and proliferation-related genes, such as *Runx2*, *ALPL*, and *BGLAP* and *MKi67* and *PCNA*, respectively, using the real-time polymerase chain reaction. In addition, the differentiation and proliferation of osteoblast cells cultured on parylene N film were validated using immunostaining. Finally, the formation of spheroids and regulation of differentiation in human mesenchymal stem cells (MSCs) on parylene N film was demonstrated. The results of this study confirm that the microenvironment with the controlled hydrophobic property of parylene N film could effectively trigger the bone differentiation and maintains the proliferation of MSCs, similar to MG-63 cells without any scaffold structures or physical treatments.

## Introduction

Parylene is a polymer composed of p-xylylene. Because of its stable physicochemical properties, parylene has been widely used for biomedical applications, including surface coatings of implants and cell culture materials^1–3^. By using parylene monomers with various functional groups, the surface properties of the parylene film can be controlled to provide different levels of hydrophilicity. Additionally, the functional groups on the parylene film surface can be modified by ultraviolet (UV) light or plasma treatments^4^. Surface-modified parylene provides different microenvironments for proliferation, as well as differentiation of osteoblast- and neuron-like cells^5,6^. For instance, for both proliferation and differentiation of PC12 cells, negatively charged oxygen-bearing surface functional groups are required for the stable attachment of dendrite-like structures^5^. In the case of osteoblast cells such as MG-63 cells, the cell cycle changes depending on the hydrophilicity of the microenvironment^4^. In this work, the osteogenic differentiation and spheroid formation potential of a parylene N film was compared with those of a UV-treated parylene N film without any scaffold structures or physical treatments.

Spheroids have been grown using various methods, including hanging drops, spinner flasks, non-adherent surfaces, and microfabricated scaffolds^7^. The formation of spheroids on a surface with controlled hydrophobicity increases cell-to-cell interactions compared with cell-to-surface interactions. Attachment of cells on the culture matrix is required for the proliferation of animal cells, and a hydrophilic surface, which can be obtained by introducing chemical functional groups, is preferable^8,9^. The surface properties of different cell types required for the formation of spheroids can be controlled by the hydrophilicity.

Osteoblast differentiation has been studied using various in vitro monolayer culture systems. Compared with the conventional monolayer cell culture system, three-dimensional (3D) cultures support osteogenic differentiation and matrix production of primary human osteoblasts and mesenchymal stem cells (MSCs) in vitro^4,7,10–12^. Generally, 3D culture systems for osteogenic cells constitute a simple micromass culture on porous sponge-like scaffolds and hydrogels containing alginate, natural polymers (e.g., chitosan), extracellular matrix components (e.g., collagen), or synthetic polymers (e.g., polylactic acid).

Because the surface properties of parylene film can be regulated using monomers with chemical functional groups and surface modifications with UV light and plasma at a controlled power, the surface properties of different cell types can be effectively controlled. In the present study, the ability of parylene N film to regulate the cellular microenvironment of osteoblast-like cells (MG-63) was determined. In particular, cell properties related to cell differentiation and proliferation were assessed by gene expression analyses and immunostaining. Finally, our findings were confirmed using human mesenchymal stem cells cultured on parylene N film.

## Results and discussion

### Formation of MG-63 cell spheroids on parylene N film

**Figure 1:**
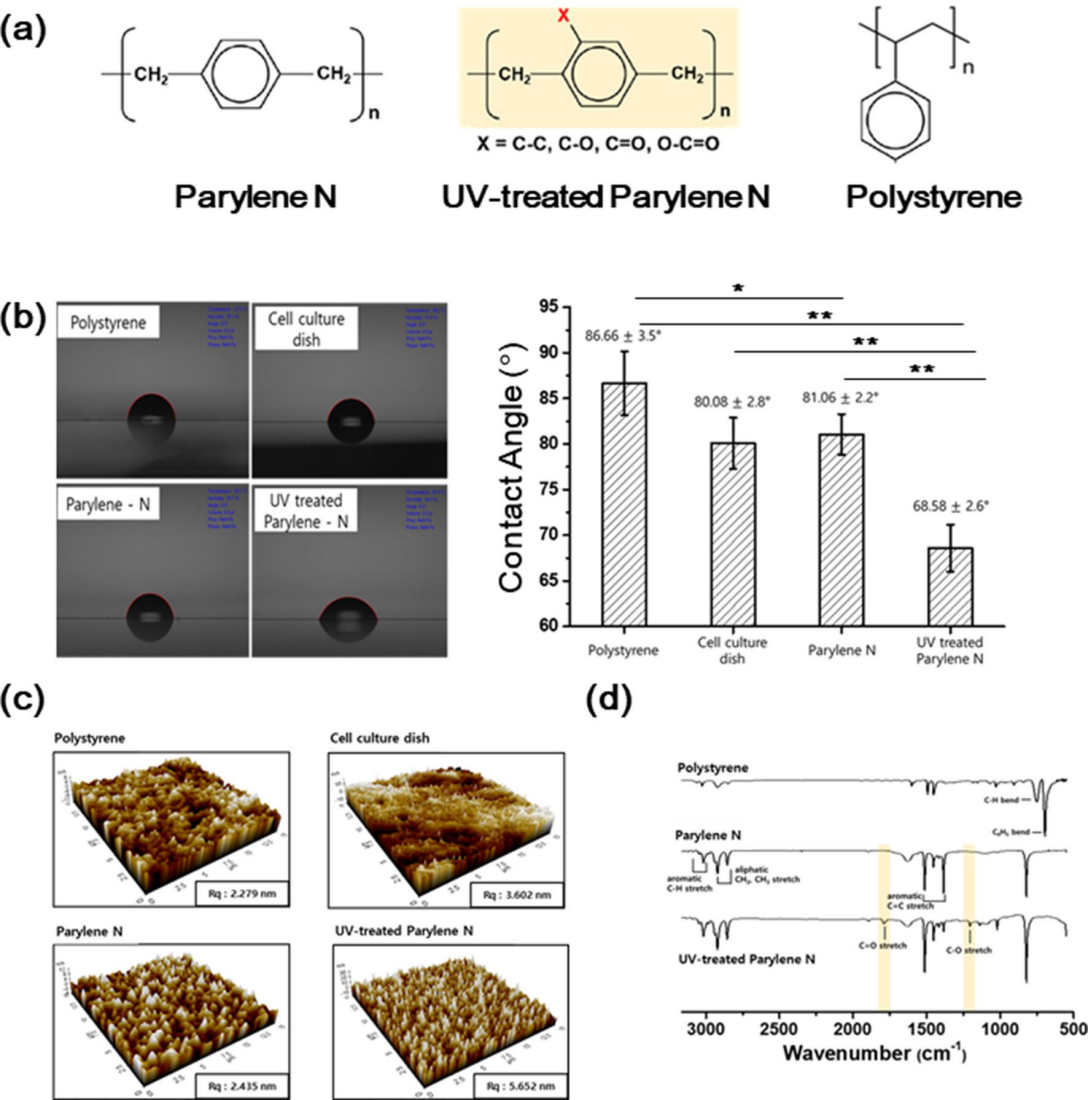
Characterization of the surface properties of the parylene N film. (**a**) Chemical structure of polystyrene, parylene N, and ultraviolet (UV)-treated parylene N. (**b**) Contact measurements with water on polystyrene, parylene N, UV-treated parylene N, and the cell culture dish (n = 5). (**c**) Surface analysis of polystyrene, cell culture dish, parylene N, and UV-treated parylene N using atomic force microscopy. (**d**) Fourier transform infrared spectra of polystyrene, parylene N, UV-treated parylene-N, and the cell culture dish. Experiments were performed in triplicate and repeated three times with similar results. ± SD. *P < 0.05; **P < 0.01.

Parylene N film was deposited thermally on a polystyrene surface. As shown in Fig. 1a, the chemical structure of the parylene N film was similar to that of polystyrene, lacking polar chemical functional groups. To characterize the surface properties of the parylene N film, the contact angle was measured and compared with those of a conventional cell culture plate, polystyrene surface, and UV-treated parylene N film.

The surface of UV-treated parylene N film contains several types of oxygen-bearing functional groups that increase its hydrophilicity^4^. The UV-treated parylene N was used for the comparison with other surfaces because this surface showed a significant influence on the cell proliferation of osteoblast- and neuron-like cells as well as the cell differentiation of neuron-like cells in the previous work^5,6^. As shown in Fig. 1b, the contact angles of the polystyrene surface, conventional cell culture plate, parylene N film, and UV-treated parylene N film were 86.7 ± 3.5°, 80.1 ± 2.8°, 81.1 ± 2.2°, and 68.6 ± 2.6° (n = 5), respectively. These results show that UV-treatment rendered parylene N’s surface hydrophilic, while the surface of the parylene N film was similarly hydrophobic to that of conventional cell culture matrices.

The surface roughness of polystyrene surfaces, conventional cell culture dishes, parylene N, and UV-treated parylene N was estimated using atomic force microscopy. As shown in Fig. 1c, the estimated average roughness ranged between 2.2 and 5.6 nm, demonstrating a similarly smooth surface for the four kinds of surfaces, in comparison with the size of cells in the microscale. As reported in previous work^5^, after UV-irradiation, the addition of oxygen species to the surface of parylene-N and parylene-C films was analyzed by XPS analysis, and the oxygen-bearing functional groups were characterized to be hydroxyl, carbonyl, and carboxylic acids, using FT-IR (Fig. 1d). These results showed that the hydrophilicity of UV-treated parylene N film was generated from the addition of such functional groups^4^.

**Figure 2:**
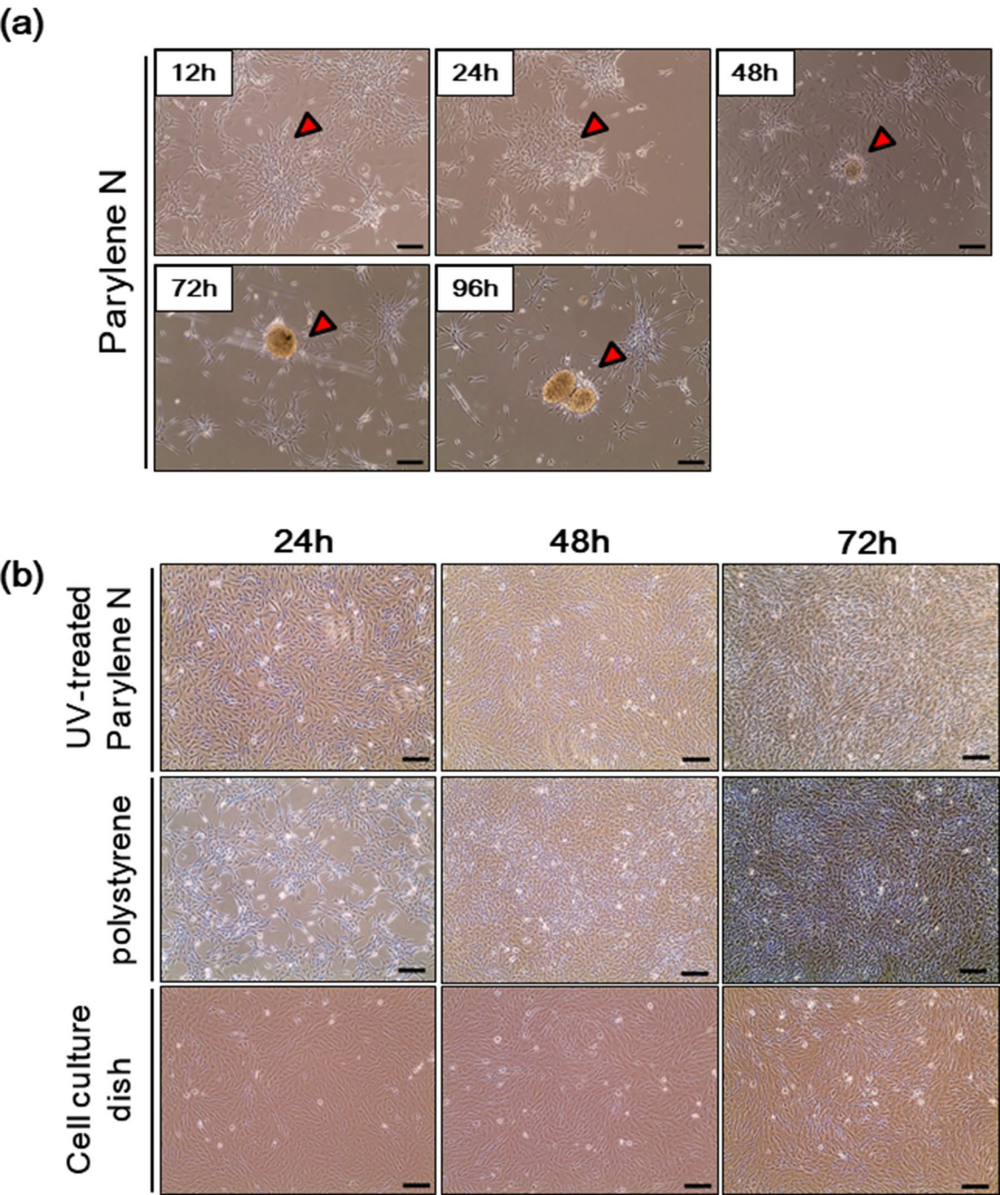
Formation of spheroids from MG-63 cells cultured on parylene N-coated film. (**a**) Osteoblast-like MG-63 cells (1 × 10^5^ cells/ml) cells were loaded on the surface of the parylene N film. After indicated incubation time, microscopic images of MG-63 cells were acquired. (**b**) Osteoblast-like MG-63 cells (1 × 10^5^ cells/ml) cells were loaded on the surfaces of UV-treated parylene N film, polystyrene, or the conventional cell culture plate. Microscopic images of MG-63 cells after indicated incubation time. Scale bar: 50 μm. Experiments were performed in triplicate and repeated three times with similar results.

As mentioned previously, spheroids form when the cell-to-cell interaction is higher than the interaction between the cell and matrix surface. A strong interaction between the cell and matrix surface can generally be observed on hydrophilic matrix surfaces with oxygen-bearing functional groups, such as hydroxyl, formyl, and carboxylic acid^13,14^. MG-63 cells were cultured on parylene N film, UV-treated parylene N film, conventional cell culture plates, and polystyrene surfaces. MG-63 cells on the parylene N film began to aggregate after incubation for 12 h, and small spheroids were observed after incubation for 24 h (Fig. 2a). Uniform-sized spheroids with diameters of ~ 100 µm were observed after incubation for 96 h. The shapes of the cells on parylene N, polystyrene, and conventional cell culture plates were compared after incubation for 24, 48, and 72 h. As shown in Fig. 2b, cells on the conventional cell culture plate, UV-treated parylene N film and polystyrene were well-attached to the surface, and spheroids did not form, even after incubation for 72 h. In the case of highly hydrophobic polystyrene, the formation of small spheroids was rare after incubation for 6 days (Sup Fig. S1).

**Figure 3:**
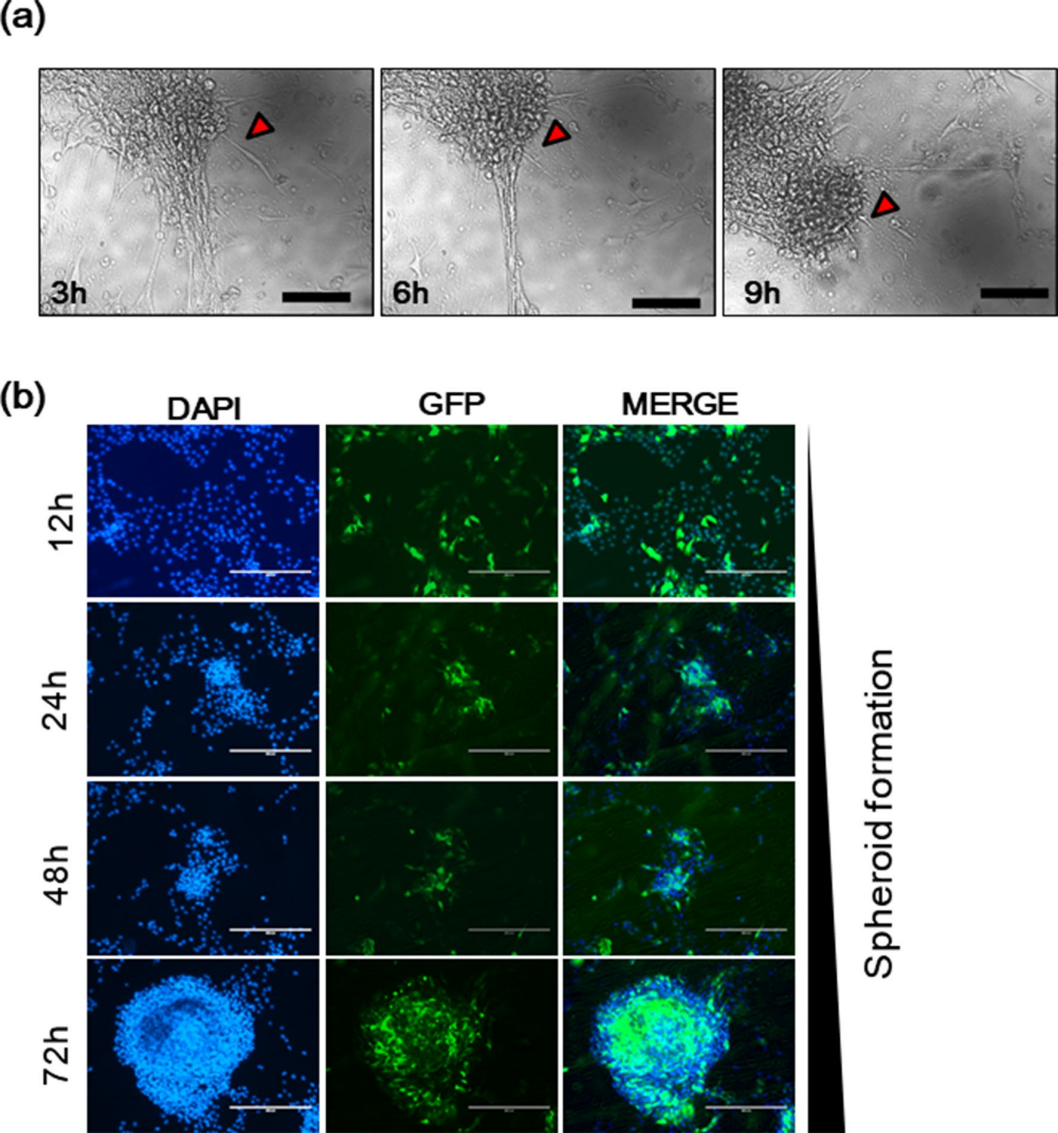
The microenvironment of parylene N-coated film enables the formation of 3D tumor spheroids from MG-63 cells. (**a**) MG-63 cells (1 × 10^5^ cells/ml) were plated on the surface of the parylene N film. After 24 h, time-lapse images of MG-63 cells on a parylene N-coated plate obtained every 3 h. Scale bar: 50 μm. (**b**) MG-63 cells (2 × 10^5^ cells/ml) were transfected with a PCMV-eGFP vector. After incubation for 24 h, GFP-transfected MG-63 cells (1 × 10^5^ cells/ml) were plated on a parylene N-coated coverslip. Fluorescence images were obtained 12 h, 24 h, 48 h, and 72 h later, followed by counterstaining with 4′,6-diamidino-2-phenylindole (DAPI). Experiments were performed in triplicate and repeated three times with similar results. Scale bar: 100 μm.

A live video showed the formation of spheroids on the parylene N film (Supplementary Material 1) and sustained a monolayer on the conventional cell culture plate (Supplementary Material 2) and polystyrene (Supplementary Material 3) for 18 h incubation time after 24 h of seeding. In the case of parylene N film, the cell edges were round, and the cores of the spheroids were moving on the surface (Fig. 3a). This morphology, i.e., round cell edges, is considered to be due to a weaker interaction between the cell and surface compared with conventional cell culture plates. This relatively stronger cell-to-cell interaction results in cell aggregation and spheroid formation. These results show that the hydrophilic surfaces of UV-treated parylene N film and conventional culture plates yield better cell attachment compared with the less hydrophilic surfaces of polystyrene and parylene N film. These results also indicate that the formation of spheroid cells can be managed by increasing the cell-to-cell interaction by controlling the hydrophilicity of the culture matrix.

Based on these preliminary findings, we carried out the same experiment with GFP-labeled MG-63 cells (Fig. 3b and Sup Fig. S2). Figure 3b shows the start of spheroid formation in a single layer of GFP-labeled MG-63 cells. The GFP signal was maintained, indirectly showing that the cells inside the spheroids were alive. Thus, we confirmed the chemical environment due to the parylene N effect influences the self-assembly of 3D spheroids from osteoblast-like MG-63 cells.

### Differentiation of MG-63 cells cultured on parylene N film

The formation of spheroids alters cell properties. According to previous studies, stem cells can be differentiated by the formation of 3D spheroids^15^. Because we studied spheroid formation on parylene N-coated film, we determined whether the cell properties changed due to the formation of spheroids. For this purpose, we performed a comparison of the global gene expression profiles of MG-63 spheroids formed on parylene N-coated plates and monolayers on the control (polystyrene) plate. The parylene N film was coated on a plate made of polystyrene. Thus, an uncoated polystyrene plate was used as a negative control for comparison of gene expression during spheroid formation. The 8,076 probe sets from spheroids cultured on parylene N that were compared with monolayers on the control plate were dysregulated (3,789 upregulated and 4,287 downregulated, Fig. S3a).

The significantly altered probes were used for hierarchical clustering and gene ontology (GO) analyses. Specifically, to gain insight into the biological significance of the 8,076 probe sets identified in our microarray analysis, we performed GO and Kyoto Encyclopedia of Genes and Genomes pathway enrichment analyses using the DAVID GO online analysis tool. The functional categories of significantly affected genes were determined. The DAVID analysis indicated that dysregulated genes primarily affected bone remodeling and regulators of bone mineralization (Sup Fig. S3b). Sonic hedgehog is also a major morphogen involved in osteoblast differentiation^16,17^. The GO analysis showed that spheroids on the parylene N-coated plate induced osteoblast differentiation.

**Figure 4:**
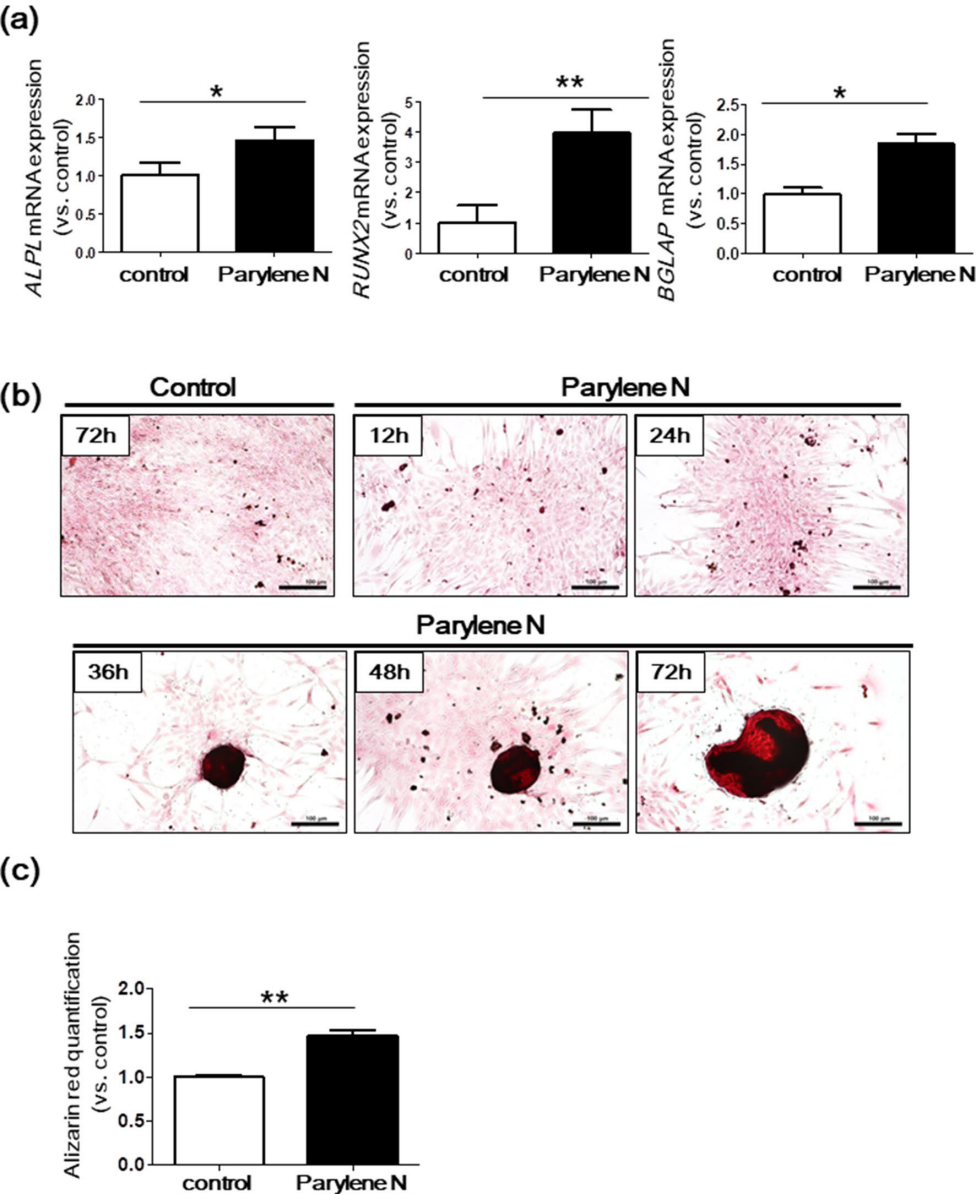
Upregulation of differentiation markers in spheroids from MG-63 cells on parylene N film. (**a**) MG-63 cells (1 × 10^5^ cells/ml) were seeded on parylene N-coated and control plates. After 72 h, *ALPL*, *RUNX2*, and *BGLAP* mRNA expression in MG-63 cells on parylene N-coated and control plates measured by the quantitative real-time polymerase chain reaction. Expression levels of the target genes were normalized to that of the housekeeping gene, *B2M,* using the 2^−ΔΔCt^ method. Data represent the mean of three independent experiments ± SD. Experiments were performed in triplicate and repeated three times with similar results. (**b**) Alizarin Red S staining of MG-63 cells (1 × 10^5^ cells/ml) grown on parylene N-coated and control plates for indicated incubation time. Scale bar: 100 μm. Experiments were performed in triplicate and repeated three times with similar results. (**c**) Quantification of Alizarin Red S was measured using MG-63 cells grown on parylene N-coated and control plates for 72 h incubation time. Data represent the mean of 6 replication ± SD. *P < 0.05; **P < 0.01.

Gene array analysis indicated that significantly dysregulated gene-encoding proteins were involved in osteoblast cell differentiation and bone morphogenesis of spheroids on parylene-N-coated film (Fig. S4). These included *RUNX2* (log2 fold change =  + 1.8) and *ALPL* (log2 fold change =  + 9.8). Osteoblast differentiation is regulated by key transcription factors, such as *RUNX2*, accompanied by the upregulation of bone matrix proteins, including *ALP* (encoded by *ALPL*)^18^. Thus, to validate the gene expression profiles, qRT-PCR was performed for selected genes. Statistically significant upregulation of *ALPL* and *RUNX2* mRNA in spheroids from the parylene N-coated plate compared with the control polystyrene plate were observed (Fig. 4a).

Osteocalcin (*BGLAP*), preferentially expressed by osteoblasts, is often used as a late marker of bone formation^19^. We observed the induction of *BGLAP* mRNA in cells cultured on parylene N, suggesting that spheroids from MG-63 cells include heterogeneous cells at various stages of osteogenic differentiation. Figure 4b,c show Alizarin Red S staining of MG-63 cells cultured on a parylene-N-coated plate and control plate. The spheroids’ staining intensity notably increased after incubation, revealing mineralization of the osteoblast-like cells^20^. Indeed, quantification of alizarin red S was significantly higher in MG-63 cells cultured on parylene N-coated plates compared to controls (Fig. 4c). Overall, these results indicate that the microenvironment of parylene N triggers the differentiation of osteoblast-like cells. Interestingly, in our study, the hydrophobicity of the surface may have been an important factor that enabled differentiation and spheroid formation of osteoblast-like cells and human mesenchymal stem cells. Analysis of the global gene expression profiles of MG-63 spheroids formed on parylene N-coated plates and monolayers cultured on a control plate indicated that dysregulated genes primarily affected calcium transport (Sup Fig. S5). For instance, *TrpC4* (+ 69.94-fold change vs. control) is a transient receptor potential cation channel protein with distinct channel properties, such as altered calcium permeability (Sup Fig. S5)^21^. The *CACNAIH* gene encodes Cav3.2, a T-type member of the α1 subunit family, a protein in the voltage-dependent calcium channel complex and mediates influx of calcium ions into cells^22,23^. Overall, genes involved in calcium import, calcium channel activity, and calcium ion homeostasis were dysregulated in spheroids formed on parylene-N-coated plates as compared to controls. Thus, transport efficiency of cations, including calcium, might be increased in spheroid formed on parylene N-coated plates. However, further studies are required to investigate the mechanisms involved in dysregulation of cation channels.

### Spheroid formation of MSCs on parylene N film

**Figure 5:**
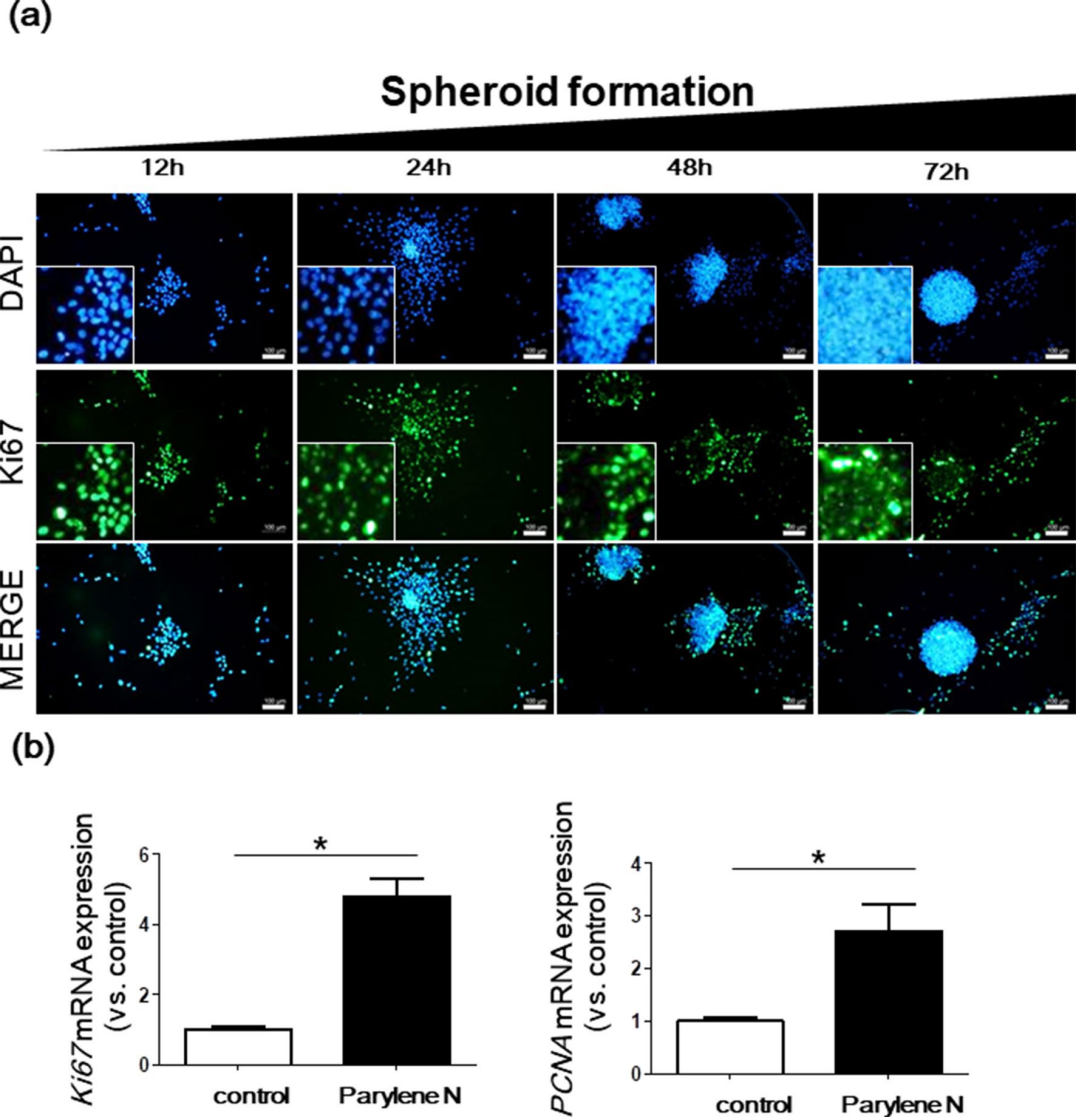
Expression of the proliferation marker, Ki67, in spheroids from MG-63 cells on the parylene N-coated plate. (**a**) MG-63 cells (1 × 10^5^ cells/ml) were cultured on parylene N-coated glass coverslips. After incubation for 12 h, 24 h, 48 h, and 72 h, and fixation, immunofluorescence images of Ki67 were obtained. Nuclei were counterstained with 4′,6-diamidino-2-phenylindole (DAPI). Scale bar: 50 μm. Experiments were performed in triplicate and repeated three times with similar results. (**b**) MG-63 cells (1 × 10^5^ cells/ml) were seeded on parylene N-coated and control plates. After 72 h, *Mki6*7 and *PCNA* mRNA levels in MG-63 cells on parylene N-coated and control plates measured by the quantitative real-time polymerase chain reaction. The expression levels of target genes were normalized to that of the housekeeping gene, *B2M,* using the 2^−ΔΔCt^ method. Data represent the mean of three independent experiments ± SD. Experiments were performed in triplicate and repeated three times with similar results. *P < 0.05; **P < 0.01.

Increased osteoblast proliferation or induced osteoblast differentiation are important keys to the enhancement of bone formation during normal bone remodeling^24^. To investigate the growth of spheroids from MG-63 cells on parylene N-coated plates, the expression of Ki67, a marker of cell proliferation, was determined by immunofluorescence staining of MG-63 cells cultured on a parylene N-coated and control coverslip (Fig. 5a and Sup Fig. S6). Cells positive for Ki67 were maintained after the spheroid formation of MG-63 cells on parylene N (Fig. 5a). Consistent with the Ki67 staining, statistically significant upregulation of the proliferation-related genes, *MKi67* and *PCNA*, mRNA was observed in the 3D spheroids compared with the control plate (Fig. 5b). Thus, in addition to differentiation, parylene-N induced the proliferation of MG-63 cells.

**Figure 6:**
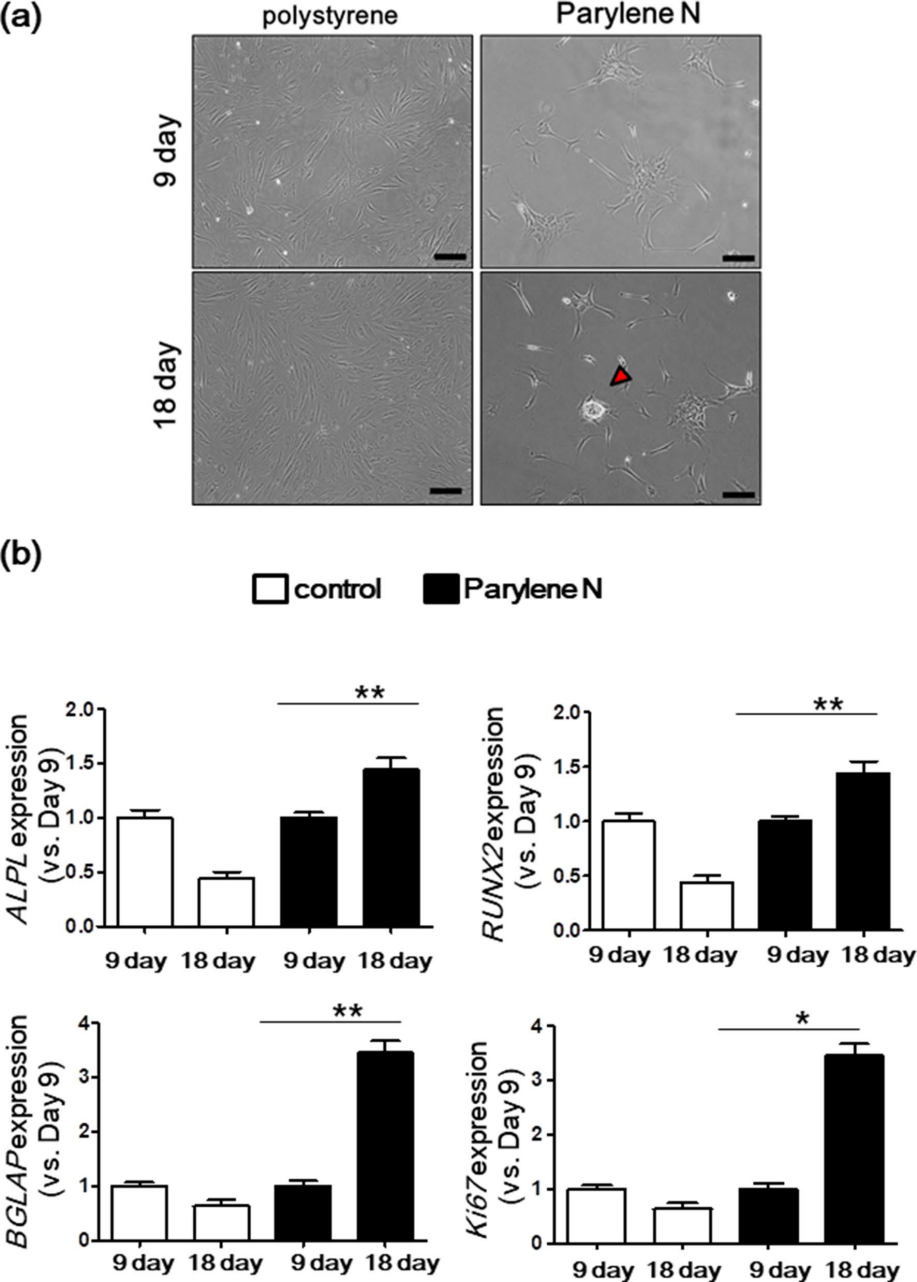
Mesenchymal stem cell (MSC) cultures on control and parylene N-coated plates. MSCs (1 × 10^5^ cells/ml) were cultured on parylene-N-coated and control plates. After 9 days and 18 days, microscopic images of MSCs were acquired. Experiments were performed in triplicate and repeated three times with similar results. Scale bar: 50 μm. (**b**) In parallel as (**a**), *ALPL*, *RUNX2*, *BGLAP*, and *MKi67* mRNA levels in MG-63 cells on parylene N-coated and control plates measured by the quantitative real-time polymerase chain reaction. Expression levels of the target genes were normalized to that of the housekeeping gene, *B2M*, using the 2^−ΔΔCt^ method. Data represent the mean of three independent experiments ± SD. Experiments were performed in triplicate and repeated three times with similar results. *P < 0.05; **P < 0.01.

Enhanced proliferation during bone formation plays a vital role in evaluating parylene N-coated plates as future injectable scaffolds for clinical bone defect repair applications. Because the microenvironment formed by parylene N promotes osteogenic differentiation of MG-63 cells, we used human MSCs that can differentiate into mesenchymal tissues, such as bone and cartilage. Similar to MG-63 cells, there were interesting morphological changes of MSCs on parylene N-coated plates. MSC morphology on the control plate was characterized by a homogenous monolayer of adherent cells. In contrast, MSCs on parylene N formed incomplete spheroids but distinct MSC colonies after 18 days of culturing (Fig. 6a). In addition, MSCs on parylene N exhibited significantly increased expression of *ALPL*, *RUNX2*, *BGLAP*, and *MKi67* mRNAs, while the control showed no change or even a reduction in the expression of these mRNAs after incubation (Fig. 6b). These results confirm that the parylene N film could effectively drive osteogenic differentiation and spheroid formation. Such results could be achieved by the control of chemical environments, through the controlled hydrophobic properties of the parylene N film. Usually, hydrophilic surfaces functionalized with oxygen species have been used for the osteogenic differentiation and spheroid formation; however, this work demonstrated that surfaces with controlled hydrophobicity could effectively drive osteogenic differentiation and spheroid formation in the absence of scaffolds or physical treatments such as hanging-drop methods.

## Conclusions

In this study, the ability of parylene N film to regulate the cellular microenvironment of osteoblast-like MG-63 cells was determined. In particular, cell properties related to differentiation and proliferation were assessed by gene expression analyses and immunostaining. Our findings were verified using human stem cells cultured on parylene N film. Our results indicate that the formation of spheroid cells can be managed by increasing the cell-to-cell interaction by controlling the hydrophilicity of the culture matrix. The results of this study confirm that the microenvironment formed by parylene N triggers the differentiation of osteoblasts and maintains the proliferation of MSCs similar to MG-63 cells.

## Material and methods

### Parylene N film deposition

The parylene N film was prepared as reported previously^4,6^. Parylene N precursors were purchased from Femto Science (Suwon Si, Gyeonggi-Do, Korea). The thickness of the parylene N film was controlled by adjusting the initial amounts of parylene N precursors. UV treatment of parylene N film was carried out using a UV lithography system at a fixed power of 23 mW/cm^2^ and wavelength of 254 nm (OS-1 k exposure; Seoul, Korea). Functional groups on the parylene film were analyzed using Fourier-transform infrared (FT-IR) spectrometry (Spectrum-100, PerkinElmer, Waltham, MA, USA) with a specular reflectance accessory (VEEMAX III, PIKE Technology, Madison, WI, USA) and liquid-nitrogen-cooled mercury–cadmium–telluride detector. For FT-IR spectroscopy analysis, 100 nm of parylene N was deposited on the gold-coating of the sample holder.

### Cell culture

Osteoblast-like cells (MG-63) were obtained from the Korean Cell Line Bank. MG-63 cells were cultured at 37 °C in 5% CO_2_ in RPMI1640 medium (Thermo Fisher Scientific, Waltham, MA, USA) consisting of 10% fetal bovine serum (FBS, v/v, Thermo Fisher Scientific) and an antibiotic solution containing penicillin–streptomycin (10,000 U/ml, Thermo Fisher Scientific). The human mesenchymal stem cell (hMSC) was kindly provided by Prof. Suh (from the department of medical engineering, Yonsei University College of Medicine, Seoul, Korea). The study was approved by the Institutional Review Board at Yonsei University Health System Severance Hospital (Seoul, South Korea). The research protocols were performed following relevant regulations and guidelines (Yonsei IRB number: 4-06-0032), and prior consent was obtained from all patients in advance. hMSC were isolated from the bone marrow as previously described^25^ and cultured at 37 °C in 5% CO_2_ in low-glucose Dulbecco’s Modified Eagle’s medium (Thermo Fisher Scientific) containing 10% FBS (v/v) and the same antibiotic solution as above. Cell images were obtained after various incubation times using an inverted microscope (Eclipse TS100, Nikon, Tokyo, Japan). MG-63 cells were plated on the parylene N-coated plate. After an incubation time of 24 h, live-cell imaging was performed using an LS620 microscope (Etaluma, San Diego, CA, USA) for 18 h. For transfection, the MG-63 cell line was plated and incubated for 24 h. Lipofectamine reagent (Invitrogen, Carlsbad, CA, USA) was used to perform green fluorescent protein (GFP) plasmid (pcDNA3.1-GFP) transfection following the manufacturer’s instructions.

### Alizarin red S staining

To measure calcium accumulation, MG-63 cells were loaded onto parylene-N-coated and control plates. After culturing for 12–72 h, the media were removed, and the cells washed with phosphate-buffered saline (PBS). Cells were fixed with 4% ice-cold paraformaldehyde (Invitrogen, Carlsbad, CA, USA) for 15 min. After removing paraformaldehyde, the cells were washed with PBS and stained with Alizarin Red S (pH 4.2) at room temperature for 10 min. Alizarin Red S was then removed, and the cells were washed with distilled water. Images were obtained using an Olympus BX53 microscope and Olympus Cell Sens software (Tokyo, Japan). To quantify Alizarin Red S, an osteogenesis assay kit (Millipore, Darmstadt, Germany) was used, following the manufacturer’s instructions. Absorbances at 403 nm were measured using a microplate reader (Molecular Devices, CA, USA).

### Quantitative real-time polymerase chain reaction (qRT-PCR)

MG-63 cells were seeded on a parylene N-coated plate or control plate. After incubating for 72 h, spheroids and cells were collected from the parylene N-coated plate and control plate, respectively. Total RNA was isolated, and qRT-PCR was performed as reported previously^26^. Gene expression levels were normalized to beta-2 microglobulin (*B2M*) mRNA expression levels in the corresponding complementary DNA samples. The following primers were used: *ALPL* forward, 5′-AAGAAAAGGGAGCACACAGG-3′, *ALPL* reverse; 5′-GCCCAAGATGACAGACGATG-3′; *RUNX2* forward 5′-TCACCTTGACCATAACCGTCT-3′; *RUNX2*reverse; 5′-TAAATCACTGAGGCGGTCAGA-3′; *BGLAP* forward 5′-CTCACACTCCTCGCCCTATTG -3′; *BGLAP* reverse 5′-CAGCCATTGATACAGGTAGCG-3′; *MKi67* forward 5′- AATCTCCACAGCCAGAGTCAT-3′; *MKi67* reverse 5′-CTCTGTGTGTGTTTGCGTAGT-3′; *B2M* forward;5′-TTACTCACGTCATCCAGCAGA-3′, and *B2M* reverse; 5′-AGAAAGACCAGTCCTTGCTGA-3′.

### Microarray datasets and analysis

The isolation of total RNA was performed as reported previously^26^. For the microarray experiment, total RNAs were isolated from MG-63 cultured on a parylene N-coated or control plate using a RNeasy Mini Kit (Qiagen, Hilden, Germany). The commercial microarray service was performed in eBiogen (Seoul, Republic of Korea). High-throughput sequencing was performed as single-end 75 sequencing using NextSeq 500 (Illumina). Genes with a two-fold change and with a *P* value less than or equal to 0.05 were chosen for further studies. Functional gene classification was assessed by GO and Kyoto Encyclopedia of Genes and Genomes pathway enrichment analyses using the DAVID GO online analysis tool (DAVID; https://david.abcc.ncifcrf.gov/).

### Immunostaining

For immunostaining, MG-63 cells were cultured on a parylene N-coated and uncoated coverslip. After incubating for 12–72 h, the cells were washed with PBS and fixed with 2% paraformaldehyde for 30 min before rewashing them with PBS. After a 3 min treatment with 0.1% Triton X-100, the coverslip was treated with 10% goat serum in PBS (v/v). Cells were stained with Ki67 (clone: SP6, Abcam, Seoul, Korea), as reported previously^4^. Images were obtained using an Olympus BX53 microscope and Olympus Cell Sens software. The percentage of Ki67-positive cells was calculated by dividing their number by the total number of cells with 4′,6-diamidino-2-phenylindole (DAPI).

### Statistical analysis

Statistical analyses were performed using GraphPad Prism Software (GraphPad Software, Inc., San Diego, CA). Comparisons between groups were performed using the t-test and the Mann–Whitney test. Results are indicated as mean ± SD; *P* values < 0.05 were considered statistically significant.

## Supplementary information

Supplementary Information.

Supplementary Video 1.

Supplementary Video 2.

Supplementary Video 3.
