# Supplementary material for: Modified parylene-N films as chemical microenvironments for differentiation and spheroid formation of osteoblast cells
Source: Sci Rep. 2020 Sep 16;10:15219. doi: 10.1038/s41598-020-71322-1 (PMC7495472; doi:10.1038/s41598-020-71322-1)

**Modified parylene N films as chemical microenvironments for differentiation and spheroid formation of osteoblast cells**

Tae-Hun Kim^a^, Jong-Sook Lee^b^, Hanhee Jo^b,c^, Yusun Park^b^, Mijin Yun^c^, Zhiquan Song^a^,

Jae-Chul Pyun^a,*^ Misu Lee^b,*^

^a^Department of Materials Science and Engineering, Yonsei University, 50 Yonsei-Ro, Seodaemun-Gu, Seoul 03722, Republic of Korea

^b^Division of Life Sciences, College of Life Science and Bioengineering, Incheon National University, Incheon, Korea

^c^Department of Nuclear Medicine, Severance Hospital, Yonsei University College of Medicine, 134 Shinchon-dong, Seodaemun-gu, Seoul 120-752, South Korea

^*^Corresponding authors:

Prof. Jae-Chul Pyun

Yonsei University, Seoul, Korea

E-mail: [jcpyun@yonsei.ac.kr](mailto:jcpyun@yonsei.ac.kr) / Tel: +82 2 2123 5851 Fax:+82 2 312 5375

Prof. Misu Lee

Incheon National University, Incheon, Korea

E-mail: [misulee@inu.ac.kr](mailto:misulee@inu.ac.kr) / Tel.: +82 32 835 8091 / Fax: + 8232 835 0754

**Figure legends**

**Supplementary Material 1.** MG-63 cells (1×10^5^ cells/ml) were plated on the parylene N-coated plate. After an incubation time of 24 h, live-cell imaging was performed using an LS620 microscope for 18 h.

**Supplementary Material 2.** MG-63 cells (1×10^5^ cells/ml) were plated on the conventional cell culture plate. After an incubation time of 24 h, live-cell imaging was performed using an LS620 microscope for 18 h.

**Supplementary Material 3.** MG-63 cells (1×10^5^ cells/ml) were plated on the polystyrene plate. After an incubation time of 24 h, live-cell imaging was performed using an LS620 microscope for additional 18 h.

**Sup Fig S1.** The number of spheroids on indicated plates. MG-63 cells (1X10^5^ cells/ml) cells were loaded on the surface indicated plates. After 6 days, microscopic images of MG-63 cells were acquired and counted the number of spheroid (>50μm).

**Sup Fig S2**. MG-63 cells (2X10^5^ cells/ml) were transfected with a PCMV-eGFP vector. After incubation for 24 h, GFP-transfected MG-63 cells (1X10^5^ cells/ml) were plated on a glass cover slip. Fluorescence images were obtained 24h, 48h and 72h later, followed by counterstaining with DAPI. Scale bar: 100 μm.

**Sup Fig S3.** (a) Scatter plots of gene expression of MG-63 cells cultured in Parylene N vs control plate. (b) Gene Ontology (GO) analysis of microarray data.

**Sup Fig S4.** The list of dysregulated genes involved in osteoblast differentiation and bone morphogenesis in MG-63 cells formed on parylene N-coated plates.

**Sup Fig S5.** The list of dysregulated genes involved in calcium transport, calcium channel activity, and calcium ion homeostasis in MG-63 cells formed on parylene N-coated plates.

**Sup Fig S6.** MG-63 cells (1X10^5^ cells/ml) were cultured on the uncoated glass cover slips. After incubation for 24h, 48h and72 h and fixation, immunofluorescence images of Ki67 were obtained. Nuclei were counterstained with DAPI. Scale bar: 50 μm.

**Sup Fig S1.**


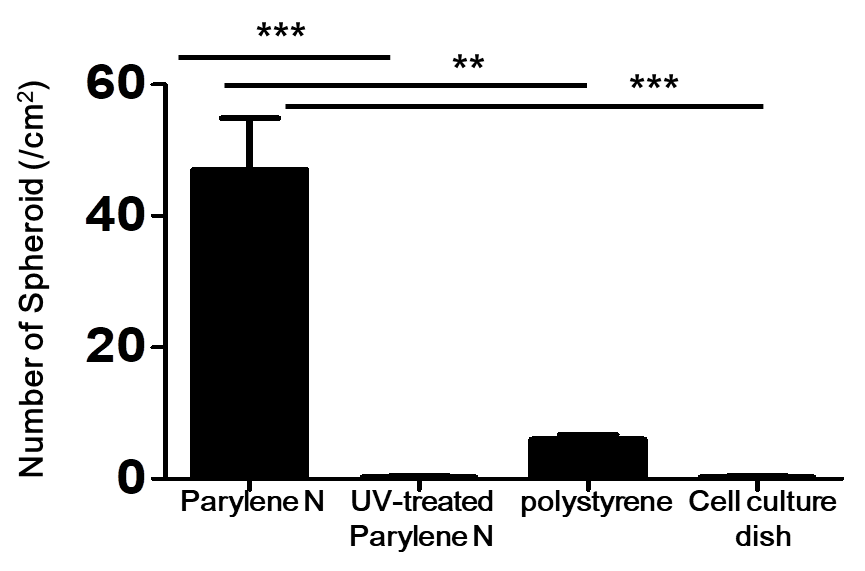


**Sup Fig S2**.


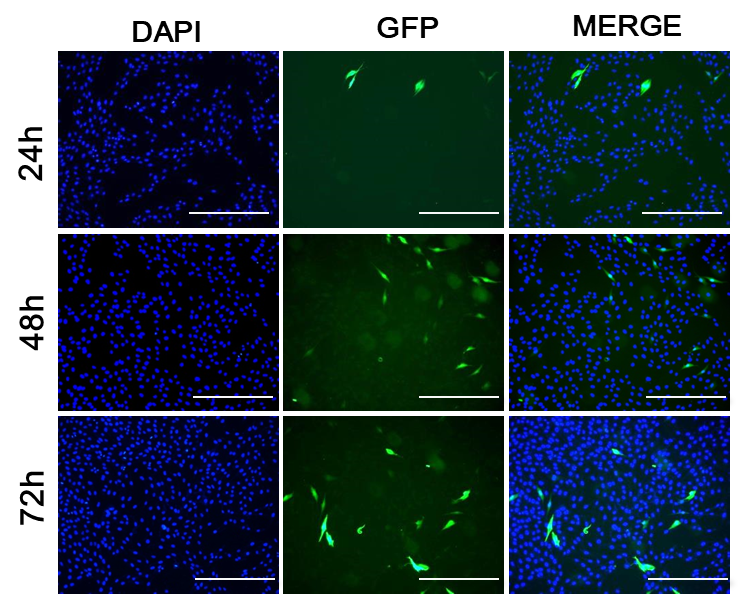


**Sup Fig S3.
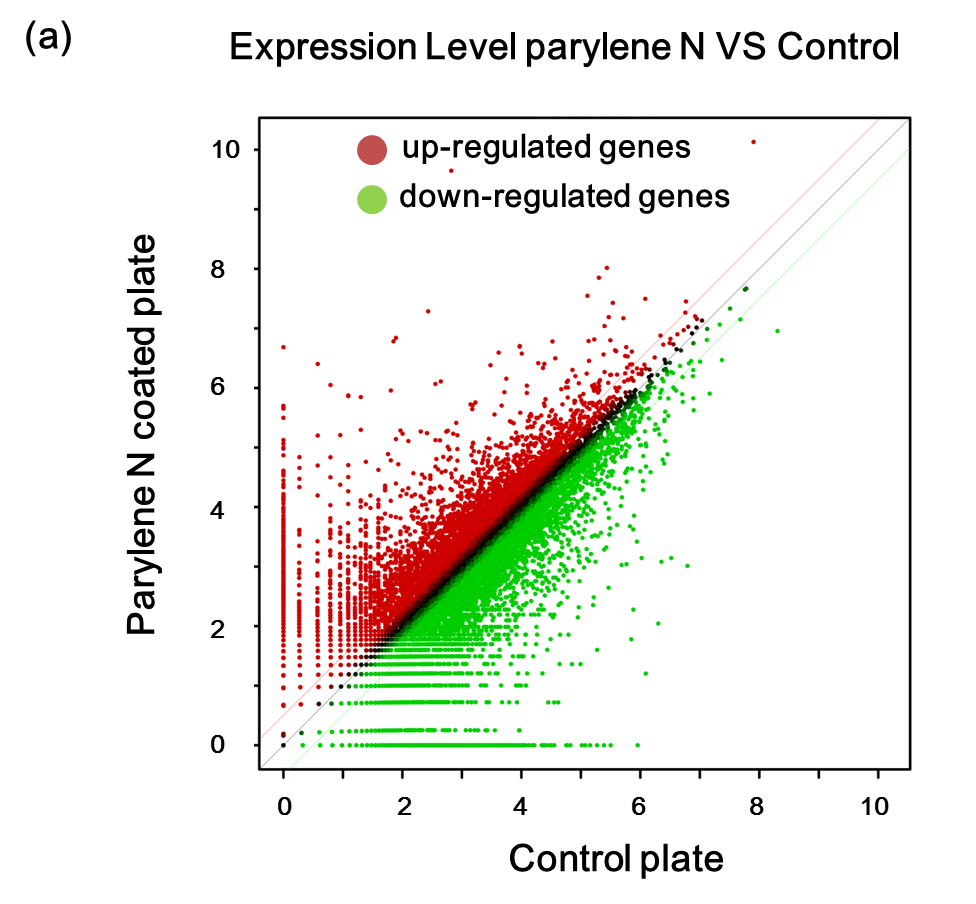
**

**
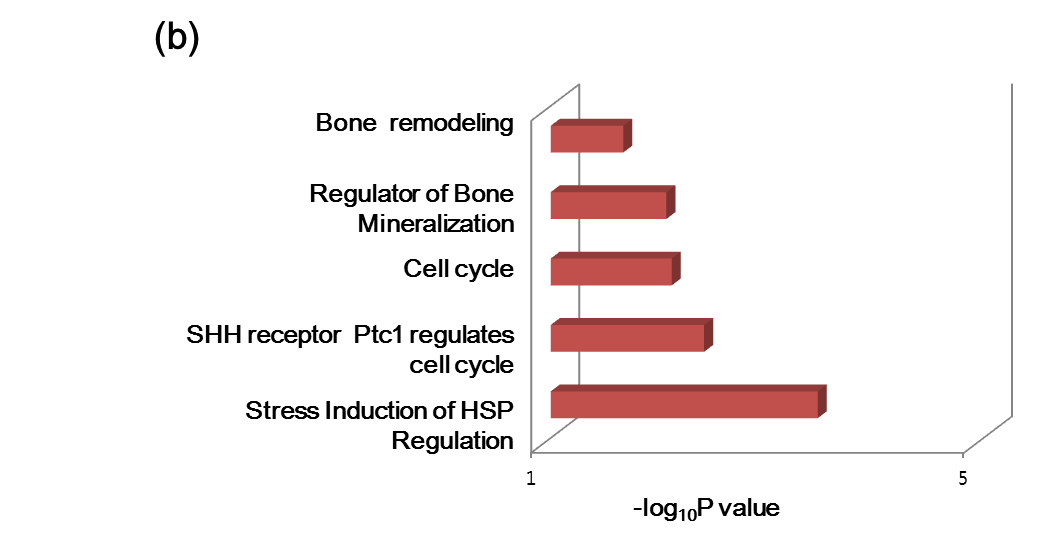
**

**Sup Fig S4.**

**
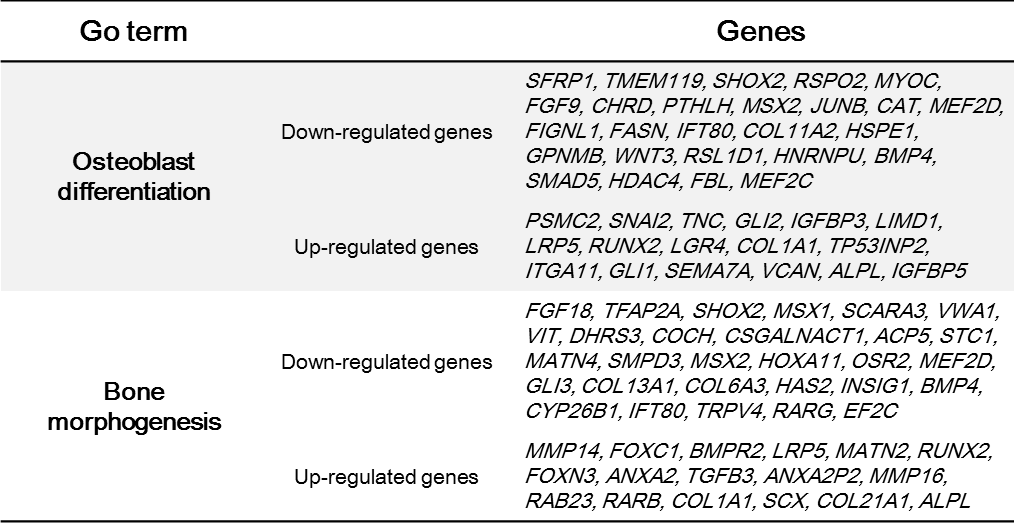
**

**Sup Fig S5.**


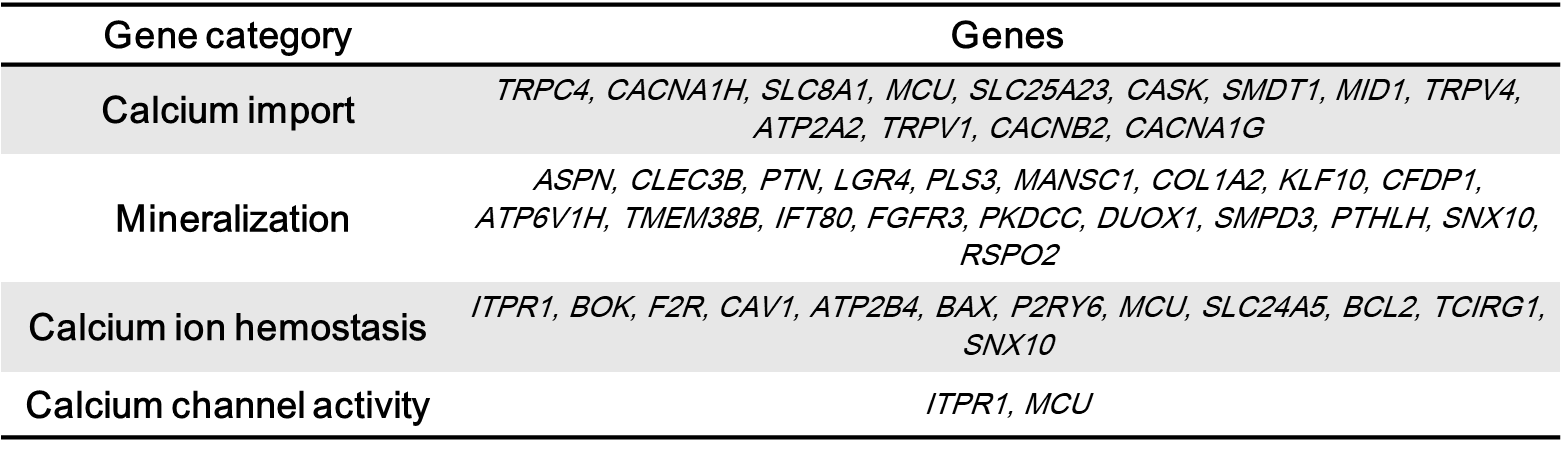


**Sup Fig S6.**


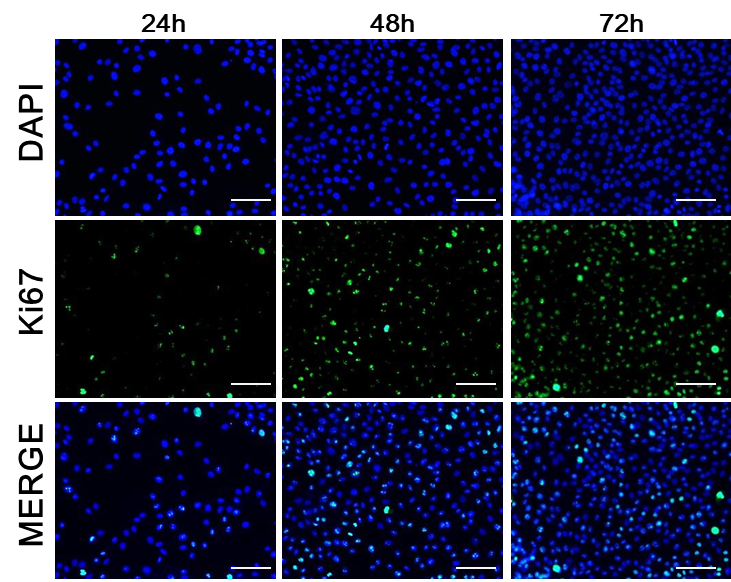

Supplement: Supplementary file 1 — Supplementary Information. [file 41598_2020_71322_MOESM1_ESM.docx]
